# COMT and ANKK1-Taq-Ia Genetic Polymorphisms Influence Visual Working Memory

**DOI:** 10.1371/journal.pone.0055862

**Published:** 2013-01-31

**Authors:** Marian E. Berryhill, Martin Wiener, Jaclyn A. Stephens, Falk W. Lohoff, H. Branch Coslett

**Affiliations:** 1 Memory and Brain Laboratory, Department of Psychology, University of Nevada, Reno, Nevada, United States of America; 2 Laboratory for Cognition and Neural Stimulation, Department of Neurology, Perelman School of Medicine of the University of Pennsylvania, University of Pennsylvania, Philadelphia, Pennsylvania, United States of America; 3 Translational Research Laboratory, Center for Neurobiology and Behavior, Department of Psychiatry, Perelman School of Medicine of the University of Pennsylvania, Philadelphia, Pennsylvania, United States of America; Yale University, United States of America

## Abstract

Complex cognitive tasks such as visual working memory (WM) involve networks of interacting brain regions. Several neurotransmitters, including an appropriate dopamine concentration, are important for WM performance. A number of gene polymorphisms are associated with individual differences in cognitive task performance. COMT, for example, encodes catechol-o-methyl transferase the enzyme primarily responsible for catabolizing dopamine in the prefrontal cortex. Striatal dopamine function, linked with cognitive tasks as well as habit learning, is influenced by the Taq-Ia polymorphism of the DRD2/ANKK1 gene complex; this gene influences the density of dopamine receptors in the striatum. Here, we investigated the effects of these polymorphisms on a WM task requiring the maintenance of 4 or 6 items over delay durations of 1 or 5 seconds. We explored main effects and interactions between the COMT and DRD2/ANKK1-Taq-Ia polymorphisms on WM performance. Participants were genotyped for COMT (Val^158^Met) and DRD2/ANKK1-Taq-Ia (A1+, A1−) polymorphisms. There was a significant main effect of both polymorphisms. Participants' WM reaction times slowed with increased Val loading such that the Val/Val homozygotes made the slowest responses and the Met/Met homozygotes were the fastest. Similarly, WM reaction times were slower and more variable for the DRD2/ANKK1-Taq-Ia A1+ group than the A1− group. The main effect of COMT was only apparent in the DRD2/ANKK1-Taq-Ia A1− group. These findings link WM performance with slower dopaminergic metabolism in the prefrontal cortex as well as a greater density of dopamine receptors in the striatum.

## Introduction

Working memory (WM) refers to the ability to maintain and manipulate information ‘on-line’ in the face of disruptions such as eye movements. Examples include remembering the number you looked up to dial or the location of your coffee mug while you continue to look at your computer. WM is studied using the full experimental toolkit including neuroimaging, investigations in participants with brain lesions, brain stimulation and behavioral tasks in normal participants. Advances in molecular genetics now make it practicable to study the underlying mechanisms of WM by looking at an individual participant's genotype. In WM the focus has been on several genes that modulate the dopamine concentration. Successful WM is believed to depend on an optimal dopamine concentration and too much or too little dopamine is considered to be detrimental to executive function (reviewed in [1], [2], [3], [4], [5], [6]. Here we investigated the effects on WM of two genes that affect dopamine activity through two single nucleotide polymorphisms that are common in the general population.

One well studied genetic polymorphism codes for two versions of the catechol-O-methyltransferase (COMT) enzyme. In the prefrontal cortex (PFC), COMT is the primary enzyme that breaks down dopamine and other catecholamines [7], [8], [9], [10]. There is a common single nucleotide polymorphism in COMT that replaces a valine with a methionine (Val^158^Met, rs4680). The rate of COMT enzymatic activity is reduced by a factor of four in the Met/Met homozygote population [11]. In other words, the efficient COMT enzyme (Val/Val) breaks down dopamine quickly leaving little dopamine in the synapse whereas the less efficient COMT enzyme (Met/Met) leaves dopamine in the synapse over a longer period of time. Behavioral findings suggest that Met/Met homozygotes perform better on a number of executive function tasks including the Wisconsin Card-Sorting Task [12], [13], [14], [15]; reviewed in [16], Complex Working Memory Span [17], and n-back WM tasks [14], [18], [19]; see also reviews in [6], [20]. Furthermore, differences become more apparent with age [21], [22], [23]. A recent meta-analysis of twenty relevant neuroimaging studies clarified the link between COMT genotype, prefrontal dopamine and cognitive task performance [24]. Across these studies the authors observed a consistent relationship (effect size of.73) between prefrontal activation and COMT genotype. However, other reports are inconsistent with these findings. For example, a recent study with 86 participants failed to find any effect of COMT in a change blindness WM study [25]. In a second study, Bruder and colleagues tested 402 participants in four WM tasks (n-back, serial position, spatial delayed response, letter-number sequencing) and only found a Met/Met benefit for letter-number sequencing [26]. Finally, a recent meta-analysis evaluating a series of cognitive tasks and COMT genotype observed no consistent relationship between performance and genotype [27]. These inconsistent findings point towards an incomplete understanding of COMT effects on cognitive performance that is compounded by task differences, the need for large numbers of participants, and perhaps most importantly by unknown interactions with other genes across multiple brain regions.

There is a parallel literature investigating polymorphisms influencing striatal dopamine. There are strong frontostriatal connections and evidence supporting a role of the striatum in higher cognition, including in WM [28], [29]. Although the striatum is classically associated with habit learning it has also been associated with updating the contents of WM [30]. Indeed, an individual's WM capacity predicts striatal dopamine synthesis [31]. Furthermore, there is experimental evidence to suggest that WM requires the basal ganglia for gating what enters WM and the prefrontal cortex for WM maintenance [29], [32], which is in accord with computational models [33], [34], [35]; see also [20]. In the striatum, D2 receptors are the most common dopamine receptor [36]. The density of D2 dopamine receptors in the striatum is influenced by polymorphisms in the DRD2/ANKK1-Taq-Ia fragment [37], [38], also referred to as the ANKK1 polymorphism. The presence of a single copy of the A1 allele (A1+) is associated with a 30–40% reduction in D2 receptor density [38]; but see [39] and reduced cognitive performance when compared to participants lacking this polymorphism (A1−). Carriers of the A1 allele perform worse on the California Verbal Learning Test of memory [40], [41] and other cognitive tasks (reviewed in [20], [42].

Thus, because dopaminergic frontostriatal pathways modulate WM performance there is good reason to investigate COMT and DRD2/ANKK1-TAQ-Ia simultaneously. The COMT Val^158^Met polymorphism dictates dopamine concentration in the PFC but not in the striatum [10]. Likewise, there are few D2 receptors in the PFC but many in the striatum [43]. One recent study explored both COMT and DRD2 effects on a series of WM tasks [17]. Stelzel et al. (2009) reported that Met/Met homozygotes performed better across WM tasks, but only when they were A1−. In short, both a slow acting dopamine-catabolizing enzyme and a high concentration of dopamine receptors were associated with good WM performance [17]. Val^158^Met and the DRD2/Ankk1-Taq-Ia polymorphisms may interact to produce differential phenotypes at the behavioral level.

In addition to WM load and genotype we were also interested in the relative contributions of COMT and DRD2/ANKK1-Taq-Ia polymorphisms with regard to interval timing. Interval timing refers to the ability to discriminate between different temporal durations. Recently, Wiener and colleagues used molecular genetics to reveal two timing circuits [44]. Participants were asked to discriminate short (500 ms) and long (2000 ms) time intervals. Response time variability increased at short intervals for the A1+ DRD2/ANKK1-Taq-Ia group and at the long intervals for the COMT Val+ group. The conclusion was that separate temporal mechanisms in the striatum and the PFC are optimized for short and long intervals, respectively. However, one unresolved issue from these findings is the cause of the disruption in timing performance. One untested possibility was that the differential response for Val+ carriers for longer durations might relate to WM load rather than timing per se. The effects for longer stimuli could simply reflect a WM deficit rather than the assumed effect of stimulus duration.

To explore these issues we investigated the effects of COMT and DRD2/ANKK1-TAQ-Ia polymorphisms on WM performance in healthy adults. In addition, we included maintenance-delay and set-size manipulations to investigate differential striatal and PFC involvement for short (1000 ms) or long (5000 ms) delays with small (4-element) or large (6-element) WM maintenance requirements. With regard to COMT, we predicted that the Met/Met homozygotes would perform significantly better than the Met/Val or Val/Val groups. These predictions were based on previous findings generally reporting superior WM performance in Met/Met participants (reviewed in [6]. Based on the work of Stelzel and colleagues (2009), we further predicted that in DRD2/ANKK1-TAQ-Ia A1−, COMT Met/Met participants, we would see better WM performance when compared to all other groups. We also investigated whether varying the WM maintenance delay would interact with participants' genotypes as demonstrated by Wiener and colleagues (2011)[44]. We had two a priori predictions. First, we predicted that COMT Val+ carriers would be disproportionately impaired at higher WM demands: longer delays and larger set sizes. Second, we predicted that the A1+ DRD2/ANKK1-TAQ-Ia carriers would be disproportionately impaired at shorter delays. Finally, set size was manipulated to avoid WM floor effects in participants with high WM capacity.

## Methods

### Ethics Statement and Participants

134 participants (mean age 22.8; standard deviation = 6.00, range = 18–57, 53 male, aged 36–57: N = 6, aged 26–35: N = 25, aged 18–24: N = 103) from the University of Pennsylvania and University of Nevada communities were recruited. Participants received payment or undergraduate course bonus credit for participation. The majority of participants were Caucasian. Participants were screened so that they had normal or corrected-to-normal vision. All participants consented to the experimental procedures and the collection of saliva for DNA analysis. The Institutional Review Boards of the University of Pennsylvania and the University of Nevada approved all experimental protocols. Participants signed informed consent documents.

### Genotyping

Saliva samples were collected with an OG-100 Oragene collection kit (DNA Genotek, Ontario, Canada), and DNA was extracted using standard Methodology. One participant did not provide a sample with sufficient DNA for analysis and a second was eliminated due to problems with sample/data labeling.

Genotyping was performed using standard Applied Biosystems ABI Taqman genotyping. Quality control included genotyping of 10% duplicates. Concordance rates were 100%.

For the COMT Val^158^Met polymorphism (rs4680), we identified 43 subjects homozygous for the Val allele, 63 Val/Met heterozygotes, and 27 Met homozygotes. This distribution was consistent with the Hardy-Weinberg equilibrium (Χ^2^ (1) = 23, p>75). For the DRD2/ANKK1-Taq-Ia (rs1800497) analysis we collapsed across A1 carriers because of low frequency of A1/A1 homozygotes (e.g. [45], awe combined A1/A1 homozygotes and A1/A2 heterozygotes as A1+ carriers that violated Hardy-Weinberg equilibrium. The results of our genotyping analysis identified 61 subjects with the DRD2/ANKK1-Taq-Ia polymorphism (A1 allele carriers: A1+), 72 subjects who lacked the polymorphism (A2 homozygotes: A1−). In the A1+ group there were 13 Met/Met, 28 Val/Met, 20 Val/Val; in the A1− group there were 14 Met/Met, 35 Val/Met, 23 Val/Val participants.

### Design

**Figure 1:**
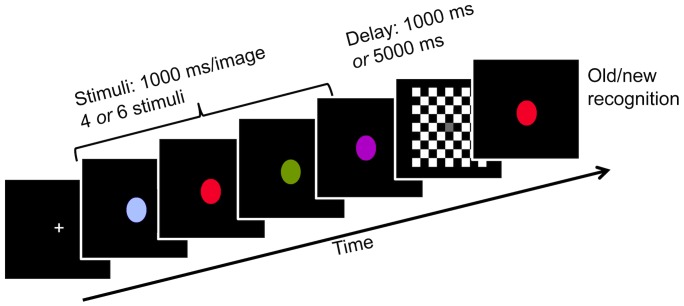
Trial sequence. After an initial fixation cross 4 or 6 stimuli were presented (1000 ms/stimulus). The WM maintenance period was either 1 or 5 s in duration and it was immediately followed by a probe image. Participants determined whether the probe image matched a previously shown stimulus. The participant initiated the next trial with a button press response.

We used a sequential presentation object WM paradigm (e.g.[46], [47], [48]. During each trial, participants viewed a series of sequentially presented circular color patches (1000 ms/stimulus); see Figure 1. Non-primary colors (e.g. peach, teal, chartreuse) were selected to avoid a verbal strategy based on over-learned labels. Trials with four or six stimuli were equally likely and pseudorandomly interleaved. Next, a checkerboard mask was presented during the variable delay duration (1000 ms or 5000 ms). Both delay durations were equally likely and unpredictable. A probe item appeared and participants made a button press response to indicate whether the probe had been presented earlier among the stimuli or not (chance = 50%). There were a total of 104 trials and sessions lasted approximately 15 minutes. Participants were instructed to respond as quickly and as accurately as possible.

### Analysis

The median correct reaction time data were included in the following analyses. To assess reaction time variability, the standard deviation for each participant for each set size and delay were also subjected to analysis. Accuracy was measured by corrected recognition (hits-false alarms). In corrected recognition measures chance is equal to 0. This measure permits calculation when the values are 0 or 1, unlike d'. The same pattern of results is expected when using corrected recognition or d' [49]. Identical analyses were also conducted using d' values (z(hits)–z(false alarms)) replacing values of 0 and 1 with. 001 and .99, respectively. All pairwise comparisons were Bonferroni corrected for multiple comparisons.

## Results

**Figure 2:**
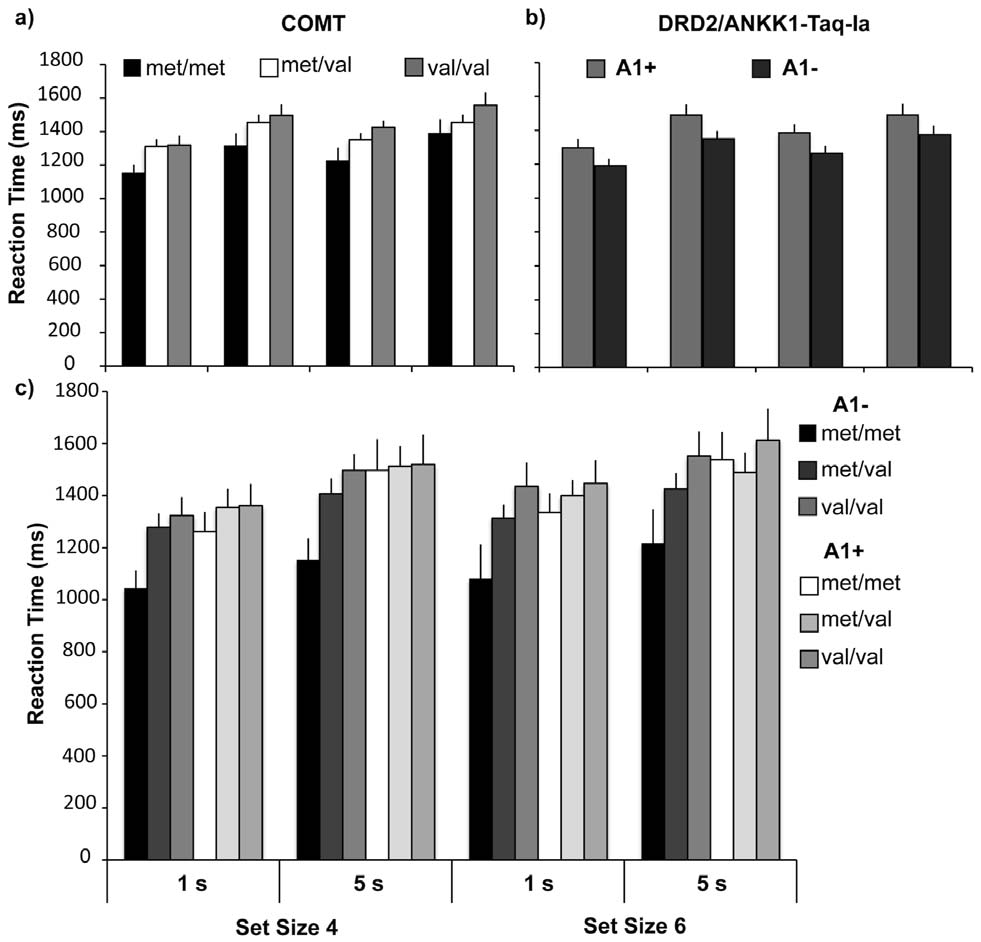
Reaction time findings. **a**, **b**) Main effects of COMT and DRD2/ANKK1-TaqIa on WM reaction time. c) The interaction of COMT and DRD2/ANKK1-TaqIa on WM performance. In each figure the data are grouped as a function of delay duration (1 s, 5 s) and set size (4, 6). Error bars represent the standard error of the mean.

The reaction time data were subjected to repeated measures ANOVA with the within-subject factors of set size (4, 6), and delay (1 s, 5 s), and the between-subjects factors of genotype: COMT (Met/Met, Met/Val, Val/Val), and DRD2/ANKK1-Taq-Ia (A1+, A1−). As expected, there was a main effect of set size (F_1, 127_ = 9.24, p = .003, partial η^2^ = 07) and delay (F_1, 127_ = 55.79, p<001, partial η^2^ = 31) such that performance was faster when there were fewer items or shorter delays. There was a main effect of COMT genotype (F_2, 127_ = 2.98, p = 05, partial η^2^ = 05); see Figure 2a. Pairwise comparisons indicated that the Met/Met homozygotes were significantly faster than the Val/Val homozygotes (M Met/Met = 1261 ms, M Val/Val = 1468 ms; p = 045) but neither group was significantly different from the intermediate Val/Met heterozygous group (M Met/Val = 1393; p's>29); see Figure 2a. There was also a significant main effect of DRD2/ANKK1-Taq-Ia genotype (F_1, 127_ = 4.60, p = 03, partial η^2^ = 03) such that the A1− group responded more quickly than the A1+ group (M A1− = 1311 ms, M A1+ = 1444 ms); see Figure 2b. None of the within- or between-subjects factors interactions, including the set size×delay or the COMT×DRD2/ANKK1-Taq-Ia interaction, approached significance (all p's>21); see Figure 2c. To assess changes in reaction time variability the standard deviations of each participant's reaction times were also subjected to the same analysis. There was a main effect of set size (F_1, 127_ = 4.31, p = 04, partial η^2^ = 03; M standard deviation set size 4 = 771.88, set size 6 = 856.19) and delay (F_1, 127_ = 15.66, p<001, partial η^2^ = 11; M 1 s delay = 723.81, 5 s delay = 904.28) such that variability increased at the higher set size and longer delay. There was a main effect of DRD2/ANKK1-Taq-Ia genotype (F_1, 127_ = 3.95, p = 05, partial η^2^ = 03) such that the A1− genotype was less variable than the A1+ genotype (M A1− standard deviation = 717.50, A1+ = 910.56), but no main effect of COMT genotype (F_2, 127_ = 2.06, p = 13) and no gene×gene interaction (F_2, 127_ = 1.87, p = 16). No other interactions approached significance (all p's>29).

Because of our a-priori assumptions regarding COMT Met/Met and A1− genotype interactions, we conducted a second set of repeated measures ANOVA in which we investigated the main effect of COMT genotype separately in the A1− and the A1+ groups. In the A1− group there were the expected main effects of set size (F_1, 69_ = 4.36, p = 04, partial η^2^ = 06) and delay (F_1, 69_ = 24.46, p<001, partial η^2^ = 26). Here, however, the main effect of COMT genotype reached significance (F_2, 69_ = 4.68, p = 012, partial η^2^ = 12). The A1− Met/Met homozygotes trended towards responding more quickly (M = 1124 ms) than the A1− Met/Val heterozygotes (M = 1356 ms, p = 07) and responded significantly more quickly than the A1− Val/Val homozygotes (M = 1452 ms, p = 01). There was no significant pairwise difference between the A1− Met/Val and the A1− Val/Val groups (p = 79). No interactions approached significance (all p's>59).

In the A1+ participants, the main effects of set size (F_1, 58_ = 4.85, p = 03, partial η^2^ = 03) and delay (F_1, 58_ = 30.38, p<001, partial η^2^ = 34) reached significance. Importantly, there was no main effect of COMT genotype in the A1+ group (F<1, p = 82) alone. None of the interactions approached significance (all p's>32).

**Figure 3:**
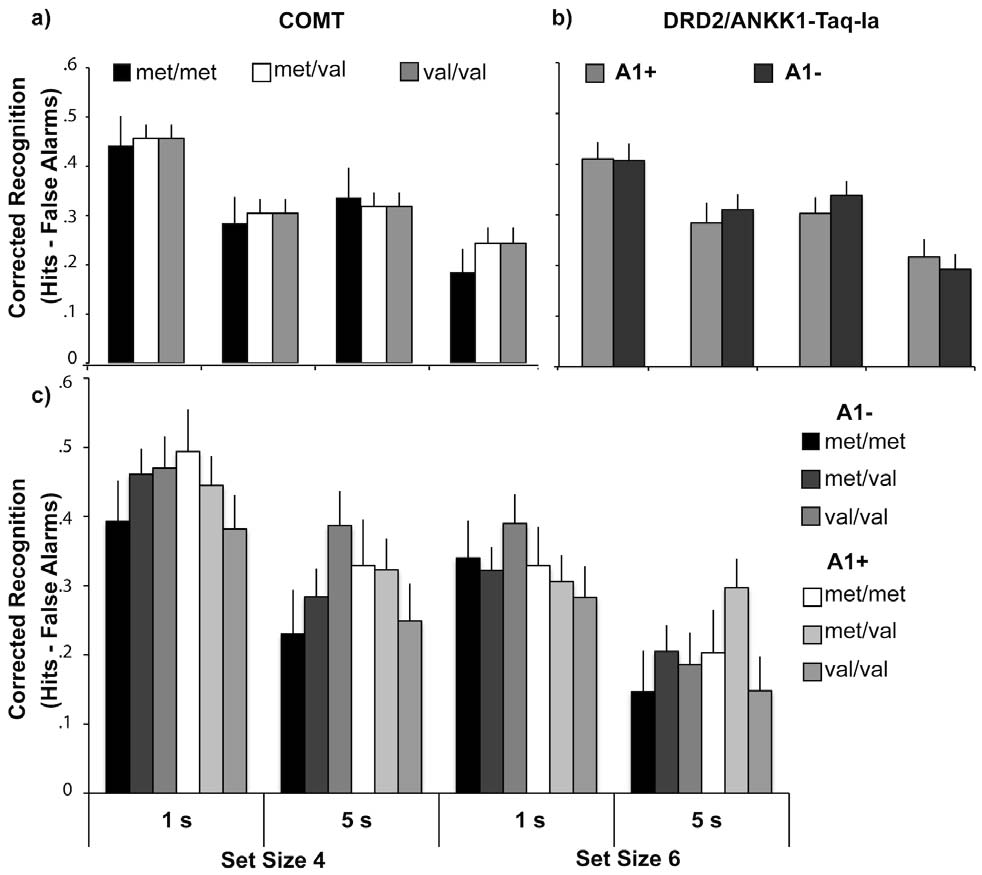
Accuracy findings. **abc**) These figures follow the same conventions as in Figure 2 but plot the accuracy (corrected recognition) data.

The accuracy measure, corrected recognition (hits–false alarms), also revealed the expected main effects of set size (F_1, 127_ = 36.92, p<001, partial η^2^ = 23) and delay (F_1, 127_ = 61.72, p<001, partial η^2^ = 33) such that performance was better when set sizes were smaller (M set size 4 = 37, 6 = 26) and delays were shorter (M 1 s delay = 38, 5 s = 25). None of the within-subjects or mixed within- and between-subject factor interactions approached significance (all p's>16). Neither was there a significant main effect of COMT or DRD2/ANKK1-Taq-Ia genotype (F's<1, p = ns). However, the interaction of COMT and DRD2/ANKK1-Taq-Ia approached significance (F_1, 127_ = 2.92, p = .057, partial η^2^ = 04). The nature of this borderline significant interaction was the following: for the A1− group, Val loading was associated with numerically greater corrected recognition performance (Mean Val/Val = 36, Met/Val = 32, Met/Met = 28). This pattern was the opposite of that observed in the reaction time data where Val loading was associated with slower performance. A detrimental effect of Val loading was observed in the A1+ group where the Val/Val group performed worse than the Met/Val or Met/Met groups (Mean Val/Val = 27, Met/Val = 34, Met/Met = 34). These trends were not apparent in repeated measures ANOVAs evaluating set size, delay and COMT genotype conducted separately on the A1− and A1+ data (COMT main effect: A1−: F_2, 69_ = 1.59, p = 21, A1+: F_2, 58_ = 1.53, p = 22); see Figure 3. Although corrected recognition is common in WM studies because it can be calculated at ceiling and floor values, we also calculated and analyzed d (z(hits)–z(false alarms)). The results remained consistent (main effects of set size: F_1, 127_ = 43.38 p<001, partial η^2^ = 26) and delay (F_1, 127_ = 59.13, p<001, partial η^2^ = 32, no main effect of COMT or DRD2/ANKK1-Taq-Ia F's<1, p = ns, or interaction COMT×DRD2/ANKK1-Taq-Ia; F_2, 127_ = 1.45, p = 24).

## Discussion

The present study confirmed that genotypes modulating dopamine concentration, COMT and DRD2/ANKK1-Taq-Ia, have main effects on WM performance. The reaction time and accuracy data provide slightly different perspectives on the nature of this influence. The reaction time data revealed that COMT genotype followed a stepwise slowing with Val loading: Met/Met<Met/Val<Val/Val. There was also a relationship between DRD2/ANKK1-Taq-Ia genotype such that carriers of the polymorphism (A1+) were slower and more variable than the non-carrier group (A1−). We also observed a numerical, but non-significant, interaction suggesting that the COMT effect was predominant in the DRD2/ANKK1-Taq-Ia A1− group. This finding provides some support for the view that there may be a degree of non-independence between these two genes. The accuracy data add complexity in interpreting these findings. A borderline significant interaction showed that behavior patterns in DRD2/ANKK1-Taq-Ia groups varied as a function of their COMT status. COMT Val loading helped the A1− group and hurt the A1+ group. Thus, we observed hints that COMT Val loading could hurt WM performance in different ways. In the A1− group it tended to slow reaction times and in the A1+ group it tended to lower accuracy. However, these epistatic effects were not sufficiently strong to produce significant gene×gene interactions and thus must be treated with appropriate caution.

### Possible Epistatic Interactions

**Figure 4:**
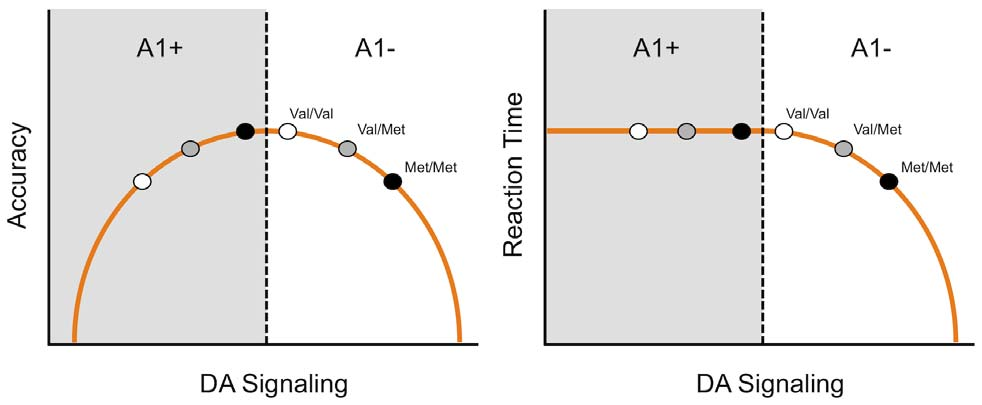
The hypothetical relationship between dopamine (DA) concentration and performance as measured by accuracy (left) or reaction time (right). The DRD2/ANKK1-TaqIa genotypes are indicated as A1+ (left panels) and A1− (right panels). COMT genotype is indicated as Val/Val (white), Val/Met (gray), or Met/Met (black).

Linking individual genotypes with performance differences is interesting, but of course genes do not operate in a vacuum and ultimately the goal is to understand how genes interact with each other. We found some suggestion of COMT and DRD2/ANKK1-Taq-Ia gene interactions. However, because there were no statistically significant gene×gene interactions any discussion remains speculative. We did observe a linear relationship between reaction time and Val loading in the A1− group but not the A1+ group. This means that when there is a low concentration of striatal D2 receptors, as in the presence of an A1 allele (A1+), reaction time was slower across all COMT genotypes. Furthermore, in A1+ participants, accuracy tends to worsen with Val loading. One possibility is that individuals with Met/Met genotypes are not at the peak of the inverted-U shaped function of optimal dopamine availability if they are also A1+; see Figure 4. Epistatic interactions may abolish the inverted-U shaped function under certain conditions, such as in the presence of the A1+ allele. To confirm this prediction future studies will need to analyze all three COMT Val^158^Met genotypes without collapsing across groups to increase power (e.g. [17].

Second, a few other studies evaluate COMT and DRD2/ANKK1-Taq-Ia genotypes. Stelzel and colleagues investigated epistatic interactions with regard to WM performance [17]. Factor analysis indicated that there was a COMT×DRD2/ANKK1-Taq-Ia interaction loading on WM manipulation but not on WM maintenance or inhibition. They concluded that COMT effects are modulated by DRD2/ANKK1-Taq-Ia genotype such that they are apparent only in the A1− group. Wishart and colleagues (2011) tested performance on the Trail Making test, in which participants connect numbers (Trails A) or alternate numbers and letters (Trails B) [50]. They observed a task×genotype interaction for the more complex Trails B task with the worst performance observed in the COMT Val+/DRD2/ANKK1-Taq-Ia A1+ group. Again, task complexity mattered. We propose that a COMT×DRD2/ANKK1-Taq-Ia interaction extends across tasks that do and do not require manipulation in WM, but these interactions become significant when manipulation is required.

The present findings support the analysis of both reaction time and accuracy measures. We observed the emergence of a speed-accuracy tradeoff selectively in A1− subjects such that those with the Met/Met genotype reacted more quickly but less accurately. This trend is consistent with the view that corticostriatal circuitry is involved in mediating speed-accuracy tradeoffs (reviewed in [51]–[52]. DRD2/ANKK1-Taq-Ia genotype determines striatal D2 levels which may alter speed-accuracy thresholds via basal ganglia output to the PFC. Thus, lower striatal D2 in A1+ participants leads to a higher threshold requirement for responses. The benefit of optimal dopamine levels in the PFC may relate to recent findings that when accuracy is emphasized sensory accumulation proceeds near-optimally [53].

### Delay Duration and Temporal Processing

We manipulated WM demands by varying the number of memoranda and the maintenance duration. The analysis revealed non-interacting main effects for both factors without observing any influence of genotype on either factor. Previous results suggest a double dissociation between COMT and DRD2/ANKK1-Taq-Ia genotypes between short, sub-second and long, supra-second duration processing [44]. The alternative possibility remained that differences in COMT groups at the supra-second range were due to different WM demands. Because the effect of COMT genotype on WM performance was independent of either set size or delay we can lay this alternative to rest and conclude that the previously reported effects of COMT on duration processing reflected differences in temporal processing. Finally, we note that there was a significant increase in reaction time variability as a function of DRD2/ANKK1-Taq-Ia genotype (A1+>A1−). This is consistent with previous reports identifying greater variability in this group at sub-second delay durations [44]. The influence of the DRD2/ANKK1-Taq-Ia genotype may thus extend beyond the sub-second durations originally reported at least when these WM task demands are imposed.

### Molecular Genetics and Synthesis

Studies looking at the molecular genetics of behavior are necessarily limited in scope. An overly simplistic trap would be to assign a ‘good’ or ‘bad’ label to a particular genotype. The COMT Val^158^Met literature provides an example case. In the cognitive literature the COMT Met/Met genotype is associated with superior executive function. However, other literatures reveal a ‘flip side’ and people with the Met/Met genotype are more vulnerable to alcoholism [54], [55]; reviewed in [56], post-traumatic stress disorder [57], and nicotine addiction (reviewed in [58]. Furthermore, Val+ individuals perform well on task switching paradigms, which require flexibility [59]; reviewed in [60], [61]. This balance between stability and flexibility may reflect the tuning of cognitive representations [62]. In summary, in the COMT Val^158^Met polymorphism the low-activity Met allele offers certain benefits to cognition and the high-activity Val allele offers others.

In contrast, there appears to be a simpler story with regard to the A1+ polymorphism of the DRD2/ANKK1-Taq-Ia gene. To date, the presence of A1+ appears to be uniformly negative. It is associated with poorer cognitive performance but it is also associated with alcoholism, problem gambling, smoking and other addictions [45]. Importantly, the question remains open as to how these isolated experimental findings can be synthesized to develop a better understanding of brain function. One challenge to this synthesis is to bring together findings from a diverse range of literatures in which the findings have been published.
